# Health anxiety is an important driver of healthcare use

**DOI:** 10.1186/s12913-022-07529-x

**Published:** 2022-02-02

**Authors:** Anja Davis Norbye, Birgit Abelsen, Olav Helge Førde, Unni Ringberg

**Affiliations:** Department of Community Medicine, UiT the Arctic University of Norway, Postbox 6050, Langnes, 9037 Tromsø, Norway

**Keywords:** Healthcare use, Health anxiety, Whiteley index, Epidemiology

## Abstract

**Background:**

Healthcare use is increasing, and health anxiety (HA) is recognized as an important associated factor. Previous research on the association between HA and healthcare use has mostly explored HA as a dichotomous construct, which contrasts the understanding of HA as a continuous construct, and compared healthcare use to non-use. There is a need for studies that examine the association between healthcare use and the continuum of HA in a general population.

**Aim:**

To explore the association between HA and primary, somatic specialist and mental specialist healthcare use and any differences in the association by level of healthcare use.

**Methods:**

This study used cross-sectional data from the seventh Tromsø study. Eighteen thousand nine hundred sixty-seven participants aged 40 years or older self-reported their primary, somatic specialist and mental specialist healthcare use over the past 12 months. Each health service was categorized into 5 groups according to the level of use. The Whiteley Index-6 (WI-6) was used to measure HA on a 5-point Likert scale, with a total score range of 0–24. Analyses were conducted using unconstrained continuation-ratio logistic regression, in which each level of healthcare use was compared with all lower levels. Morbidity, demographics and social variables were included as confounders.

**Results:**

HA was positively associated with increased utilization of primary, somatic specialist and mental specialist healthcare. Adjusting for confounders, including physical and mental morbidity, did not alter the significant association. For primary and somatic specialist healthcare, each one-point increase in WI-6 score yielded a progressively increased odds ratio (OR) of a higher level of use compared to all lower levels. The ORs ranged from 1.06 to 1.15 and 1.05 to 1.14 for primary and somatic specialist healthcare, respectively. For mental specialist healthcare use, the OR was more constant across levels of use, ranging between 1.06 and 1.08.

**Conclusions:**

In an adult general population, HA, as a continuous construct, was significantly and positively associated with primary, somatic specialist and mental healthcare use. A small increase in HA was associated with progressively increased healthcare use across the three health services, indicating that the impact of HA is more prominent with higher healthcare use.

**Supplementary Information:**

The online version contains supplementary material available at 10.1186/s12913-022-07529-x.

## Background

Internationally, healthcare use is increasing. The main reasons for healthcare use are symptoms of illness and disease. However, there is a growing concern about the trend in over-diagnosis and over-treatment [1]. There are several reasons for this trend, but both patient preference and patient wishes for reassurance account for a considerable number of referrals [2, 3]. In Norway, general practitioners (GPs) are gatekeepers for treatment in both somatic and psychiatric specialist healthcare. Thus, patients must be referred, mainly by GPs, to be entitled to care in specialist health services. Approximately 14% of all consultations in primary healthcare in Norway lead to referrals for specialist healthcare [4].

Fear and anxiety may have an impact on perceived illness and therefore on the need for contact with health services, known as health anxiety (HA). Although there is no consensus for a definition of HA [5], it was first suggested as a milder form for hypochondriasis commonly assessed in a non-psychiatric setting [6]. At present, HA is seen both as conceptually different than hypochondriasis [5, 7], and as a milder form of the diagnosis of hypochondriasis, describing the continuum of worry and anxiety [6] and also the diagnosis of hypochondriasis is being evaluated with alterations both in the diagnostic manuals DSM-V [8] and ICD-11 [9]. In concordance with other authors [10, 11], we conceptualize health anxiety as a condition including hypochondriasis in its most serious form, but which is not limited to the diagnostic criteria.

Although HA is thought to be a continuous construct [11], most studies have studied HA as a categorical construct, dichotomizing HA into severe HA or no/little HA. The prevalence of severe HA in the general population varies from 2 to 10% [12] but has been reported to be as high as 78% in patient populations [13], and one review reported that the prevalence of severe HA is increasing in student populations [14]. There are several negative consequences of HA. Previous research has found associations between HA and higher levels of health impairment [15] and shown that HA increases the risk of long-term sick leave [16]. Severe HA is also an independent risk factor for disability pensions [15]. In a large Norwegian cohort, HA increased the risk of ischaemic heart disease by 70% after cardiovascular risk factors were accounted for [17].

### The association between health anxiety and healthcare use

Severe HA is recognized as an important driver of increased healthcare use due to reassurance-seeking behaviour [18]. Frequent attenders in both primary and specialist care have higher HA scores [19–22], and HA commonly is observed alongside physical and mental morbidity [23]. However, one study found no association between healthcare use and increasing HA severity [24], while another found that people with mild HA used primary care significantly less often than people with a medical condition [13]. To explore the association between different levels of HA and healthcare use, studies must be performed in a general population.

### The knowledge gap and our aim

A common feature of the existing literature is the dichotomization of HA, healthcare use [25–28] or both [23, 29, 30]. In addition, few studies have explored how HA is associated with the use of different healthcare services. Two studies explored different health services but reported overall healthcare use without differentiating between the services [25, 29]. Sunderland et al. [23] reported information on both consultation with GPs and mental health professionals; however, the reason for attendance was reported as “due to a mental problem”. This might lead to an underestimation of the association, as people with HA often perceive their symptoms to be somatic rather than psychological in character [12].

Only Bobevski et al. [28] reported the association between HA and the use of primary healthcare, psychiatrist or psychologist healthcare and medical specialist healthcare; however, they still reported HA as a dichotomous construct. To our knowledge, only Tomenson et al. [31] explored the level of HA and different levels of use in a general population and found that HA was a predictor of increased healthcare use; however, they examined only primary care.

We therefore aimed to determine how HA, as a continuous construct, was associated with primary, somatic specialist and mental specialist healthcare use and to explore any differences in the potential association by level of healthcare use. We hypothesized that HA and primary healthcare use would be most strongly associated due to self-initiated consultations, whereas somatic and mental specialist healthcare use would be less affected by increasing HA due to the gatekeeper function of primary healthcare.

## Methods

### Study design and population

This study used cross-sectional, self-reported data from the Tromsø study: Tromsø 7, which was conducted in 2015–2016. The Tromsø study is a large Norwegian population-based health survey; see information about the Tromsø study and the data collection described elsewhere [32, 33]. In Tromsø 7, all inhabitants in the municipality of Tromsø aged 40 years or older were invited to participate, for a total of 32,591 men and women. By the end of 2016, 21,083 participants had taken part in Tromsø 7, resulting in a response rate of 65%.

### Variables

#### Outcome variables

The respondents answered questions related to healthcare use by reporting whether they had consultations with different health services or admissions to hospital during the past 12 months and the number of consultations. Healthcare use was divided into three main categories. Primary healthcare use included consultations with a GP or an emergency ward. Somatic specialist healthcare use included admissions to hospital, consultations with a somatic outpatient hospital service or medical specialist in private practice. Mental specialist healthcare included consultations with a psychiatric outpatient hospital service or consultations with a psychologist or psychiatrist in private practice.

Each of the three outcome variables was divided into five categories, where 0 represented non-use and 1–4 represented quartile levels of successively increasing numbers of consultations among users.

#### Exposure variable

We measured HA using the six-item Whiteley Index-6 (WI-6), which has shown satisfactory psychometric properties [34]. This index has 5-point Likert scale response options, and the item scores are summed to a total score ranging from 0 to 24.

Table 1 provides an overview of the WI-6 questions. All respondents answered each question with one of the following response options: “not at all”, “to some extent”, “moderately”, “to a considerable extent” or “to a great extent”. The item scores were accordingly transformed into values from 0 to 4 and summed to a total score. The WI-6 score is also presented as a 5-category variable, where 0 = not at all and 1–4 denote quartiles 1–4.

**Table 1:** Questions included in the WI-6

Question	Text
1	Do you think there is something seriously wrong with your body?
2	Do you worry a lot about your health?
3	Is it hard for you to believe the doctor when he tells you there is nothing to worry about?
4	Do you often worry about the possibility that you have a serious illness?
5	If a disease is brought to your attention (e.g., via TV, radio, internet, newspapers or someone you know), do you worry about getting it yourself?
6	Do you find that you are bothered by many different symptoms?

#### Confounders: morbidity, demographic and social variables

We included three groups of possible confounders in the analyses, as they were believed to be associated with both the level of HA and healthcare use.

##### Morbidity

Both mental and physical illnesses are the main reasons for healthcare use and have previously been found to confound the association between severe HA and healthcare use [23, 28, 31]. We used one variable for physical illness and two for mental illness. The participants reported whether they had any of the following conditions: high blood pressure, heart failure, atrial fibrillation, angina pectoris, diabetes, kidney disease, chronic bronchitis/emphysema/chronic obstructive pulmonary disease, asthma, cancer, rheumatoid arthritis, arthrosis, migraine and previous myocardial infarction or stroke. These self-reported diseases were merged into one variable called “physical illness” and categorized as none, one, or two or more, independent of the type of illness, in line with Tomenson et al. [31].

Mental illness was reported in two different ways. For analyses regarding primary healthcare and somatic specialist healthcare use, we included the question “Have you ever had, or do you currently have, psychological problems for which you have sought help?” The response options included “no”, “yes, now” and “yes, previously”. Due to multicollinearity between this question and the use of mental healthcare, the measurement tool Hospital Anxiety and Depression Scale (HADS) [35] was included as an indicator of mental illness in the analyses of mental healthcare use. The HADS is a questionnaire based on participants’ responses to 14 questions concerning symptoms of anxiety or depression the past week. The HADS cut-off was set at 15 points out of 42 [35].

##### Socioeconomic variables

Both education and income have been found to be associated with both HA and healthcare use, but with different trends for different types of health services [36]. The participants were asked to report their education as “the highest level of education you have completed”, with four categories: primary education up to 10 years of schooling, vocational/upper secondary education (minimum 3 years), college/university (< 4 years) or college/university (≥ 4 years). Household income was reported according to four categories: low (less than NOK 451000, approximately 12,000 British pound sterling (GBP), lower middle (NOK 451–750,000), upper middle (NOK 751000–1 million) or high (more than NOK 1 million, approximately 80,000 GBP).

Previous research [23, 26, 32] has found social factors to be related to the level of HA. “Do you live with a spouse/partner?” was reported as “yes” or “no”. Due to their large correlation, two questions about the quality of friendship (“Do you have enough friends who can give you help and support when you need it?” and “Do you have enough friends you can talk confidentially with?”) were merged into a variable named “Quality of friendship”, which included three categories: no, for those who answered “no” to both original questions; to some extent, for those who answered “yes” to only one original question; and yes, for those who answered “yes” to both original questions. Finally, the participants reported their participation in organized activity with the following options: “never or just a few times a year”, “1-2 times a month”, “approximately once a week” or “more than once a week”.

##### Demographic variables

The demographic variables included gender and age as of 31.12.2015.

### Statistical analysis

All analyses were performed with STATA version 16.1 (STATA Corp LP, College Station, Texas, USA). Participants were excluded if they had missing or invalid responses to the outcome variables or the exposure variable. As a sensitivity analysis, we repeated all analyses for participants who also had complete responses to all confounders. Since there were no changes in the results, we include participants with complete responses to the exposure (HA) and outcome variables in the results section.

In the descriptive analyses, means (medians) were calculated for the continuous variables, and frequency distributions were calculated for the categorical variables. The associations between HA and different levels of healthcare use are presented as summary plots. HA was included as an exposure variable in all regression analyses and supplemented with relevant confounders. The analyses were conducted in a stepwise manner; we first presented an unadjusted model, then a model adjusted for morbidity, and finally a third model adjusted for all relevant confounders. The level of statistical significance (*p*-value) was set at 0.05.

As the proportional odds assumption was not met for the ordinal regression for either outcome variable, we used unconstrained continuation-ratio regression analysis [37] to model healthcare use. The unconstrained continuation-ratio model compared each level of healthcare use with all lower response levels and allowed the odds ratio (OR) to vary for each comparison [37]. The ORs were thus interpreted as threshold-specific exposure effects [38], where the effect of exposure (X) depended on the category (Y).

We explored possible interactions between morbidity and healthcare use by adding an interaction term in the regression model and performed stratified analyses where applicable.

### Ethics

The study was conducted in accordance with the Declaration of Helsinki and was approved by the Regional Committee for Medical and Health Research Ethics (ID 2016/1793). All participants gave written informed consent before admission.

## Results

### Participant characteristics and descriptive statistics of healthcare use in the study population

A total of 21,083 persons aged between 40 and 99 years participated in this study; 52.5% were women, and the mean age was 56 (SD 11) years. After excluding participants with missing or invalid responses for the outcome or exposure variables, 18,499, 18,311 and 18,158 participants reported whether they had used primary healthcare, somatic specialist healthcare and mental healthcare, respectively, during the last 12 months. The distribution of users was different across the three healthcare services; 80% reported having consultations in primary healthcare the past 12 months, whereas 40% reported having consultations in somatic specialist healthcare, and 4% reported having consultations in mental specialist healthcare. The frequency distribution of participants and number of consultations are presented in Table 2.

**Table 2:** Frequency distribution of healthcare utilisation (primary, somatic specialist and mental specialist healthcare use), and associated health anxiety measured by WI-6, mean(median) values

Variable				Percent	HA by WI-6, mean(median)
	Categories	Levels of use	N		
Primary healthcare (PHC)	Non-use		3753	20%	1.9 (1)
	PHC users:	1st level (1 consultation)	3904	21%	2.3 (1)
		2nd level (2 consultations)	3558	19%	2.9 (2)
		3rd level (3–4 consultations)	3706	20%	3.6 (3)
		4th level (5–89 consultations)	3578	20%	4.9 (4)
	Total		18,499	100%	
Somatic specialist healthcare (SSHC)	Non-use		11,050	60%	2.6 (2)
	SSHC users:	1st level (1 consultation)	2902	16%	3.1 (2)
		2nd level (2 consultations)	1841	10%	3.7 (3)
		3rd level (3 consultations)	828	5%	4.0 (4)
		4th level (4–170 consultations)	1690	9%	4.9 (4)
	Total		18,311	100%	
Mental specialist healthcare (MSHC)	Non-use		17,517	96%	2.9 (2)
	MHC users:	1st level (1–3 consultations)	202	1%	5.1 (4)
		2nd level (4–6 consultations)	146	1%	4.7 (4)
		3rd level (7–12 consultations)	148	1%	5.1 (5)
		4th level (13–130 consultations)	145	1%	6.2 (6)
	Total		18,158	100%	

Among the users of either healthcare service, the median numbers of consultations were 2 consultations for primary and somatic specialist healthcare and 6 consultations for mental healthcare in the past 12 months. The mean (median) WI-6 score for all participants was 3.1 (2) out of 24 points, with a mode of 0. The mean (median) numbers of contacts with primary healthcare, somatic specialist healthcare and mental specialist healthcare in the last year by HA, social and demographic variables and somatic and mental morbidity are outlined in Table 3. In categorizing HA according to the quartiles of the WI-6 score, we found that healthcare use increased with increasing HA, especially in primary and somatic specialist healthcare.

**Table 3:** Mean (median) number of consultations last year in primary healthcare (PHC), somatic specialist healthcare (SSHC) and mental specialist healthcare(MSHC) according to health anxiety, social and demographic variables and somatic and mental morbidity

Variable				PHC use, mean (median)	SSHC use, mean (median)	MSHC use, mean (median)
	Categories	N	Percent			
WI-6 score of HA	0 points	5162	26%	1.8 (1)	0.7 (0)	0.2 (0)
	1. quartile (1–2 points)	5564	28%	2.4 (2)	0.9 (0)	0.2 (0)
	2. quartile (3 points)	2048	10%	2.9 (2)	1.1 (0)	0.3 (0)
	3. quartile (4–6 points)	4645	23%	3.4 (2)	1.4 (0)	0.4 (0)
	4. quartile (7–24 points)	2847	15%	5.0 (4)	2.4 (1)	1.1 (0)
		20,266				
Age	40–49 years	6432	32%	2.7 (2)	1.1 (0)	0.6 (0)
	50–59 years	6035	30%	2.8(2)	1.1 (0)	0.4 (0)
	60–69 years	5179	24%	2.9 (2)	1.2 (0)	0.2 (0)
	70–79 years	2676	11%	3.4 (2)	1.4 (0)	0.04 (0)
	80 years or older	761	3%	4.3 (3)	2.0 (0)	0.1 (0)
	Total	21,083				
Gender	Female	11,074	51%	3.2 (2)	1.3 (0)	0.5 (0)
	Male	10,009	49%	2.5 (2)	1.0 (0)	0.2 (0)
	Total	21,083				
Educational level	Primary/partly secondary education	4796	23%	3.5 (2)	1.1 (0)	0.3 (0)
	Upper secondary education	5756	28%	3.0 (2)	1.2 (0)	0.3 (0)
	Tertiary education, short	4008	19%	2.9 (2)	1.2 (0)	0.4 (0)
	Tertiary education, long	6145	30%	2.4 (2)	1.2 (0)	0.5 (0)
	Total	20,705				
Household income	Low (less than NOK 451.000)	4545	23%	3.7 (2)	1.5 (0)	0.6 (0)
	Lower middle (NOK 451–750.000)	5884	29%	3.1 (2)	1.3 (0)	0.4 (0)
	Upper middle (NOK 751–1 million)	4741	23%	2.7 (2)	1.1 (0)	0.3 (0)
	High (More than NOK 1 million)	5015	25%	2.1 (1)	1.0 (0)	0.2 (0)
	Total	20,185				
Physical illness	None	10,924	52%	2.0 (1)	0.8 (0)	0.3 (0)
	One	6171	29%	3.3 (2)	1.4 (0)	0.4 (0)
	Two or more	3987	19%	4.8 (4)	2.2 (1)	0.4 (0)
	Total	21,082				
Mental illness	No	17,660	87%	2.6 (2)	1.1 (0)	N/A
	Yes, now	898	4%	6.0 (5)	1.9 (0)	
	Yes, previously	1826	9%	3.6 (3)	1.5 (0)	
		20,384				
HADS	Under 15 points	17,864	93%	N/A	N/A	0.2 (0)
	15 points or more	1268	7%			2.5 (0)
	Total	19,132				
Living with a spouse/partner	No	4609	23%	3.2 (2)	1.3 (0)	0.6 (0)
	Yes	15,283	77%	2.7 (2)	1.2 (0)	0.3 (0)
	Total	19,892				
Do you have friends you can get support from and talk confidentially with? (“Quality of friendship”)	No	1621	8%	3.7 (2)	1.4 (0)	1.2 (0)
	To some extent	1774	9%	3.2 (2)	1.3 (0)	0.5 (0)
	Yes	17,117	83%	2.7 (2)	1.2 (0)	0.3 (0)
	Total	20,512				
Participating in organised activity	Never, or just a few times a year	11,310	5%	3.0 (2)	1.2 (0)	0.4 (0)
	1–2 times a month	4981	24%	2.9 (2)	1.3 (0)	0.3 (0)
	Approximately once a week	2597	13%	2.7 (2)	1.2 (0)	0.3 (0)
	More than once a week	1856	9%	2.5 (2)	1.0 (0)	0.4 (0)
	Total	20,744				

### The association between health anxiety and healthcare use

**Fig. 1:**
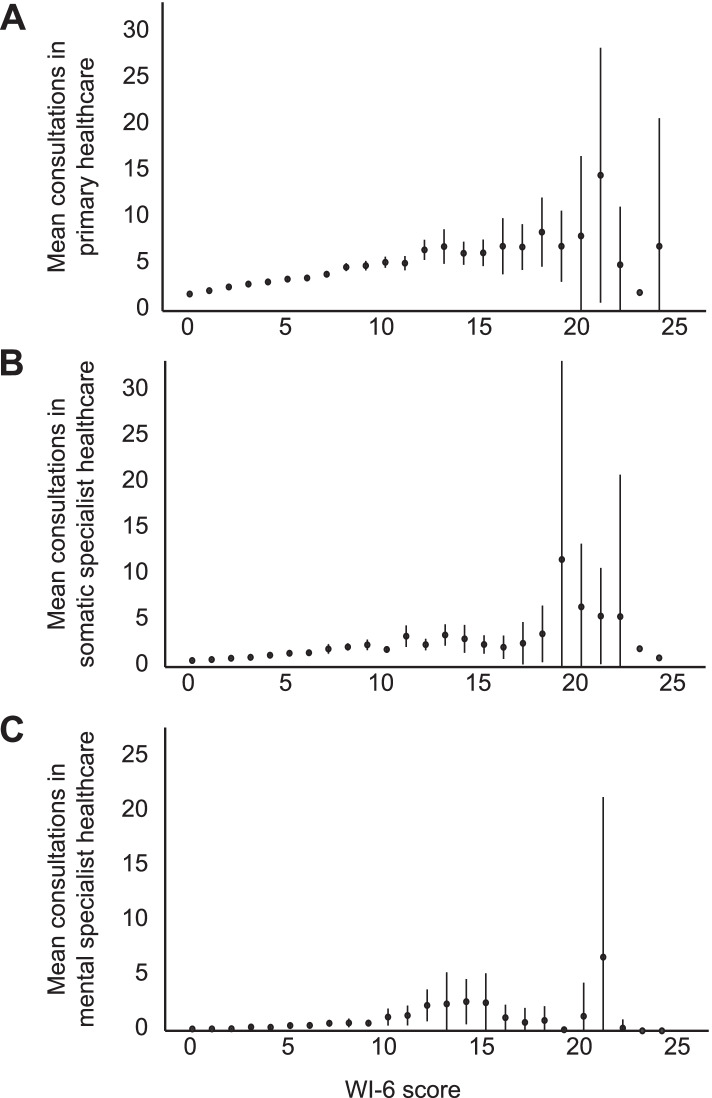
The association between the exposure variable HA and healthcare use for the outcome variables primary healthcare (**a**), somatic specialist healthcare (**b**) and mental specialist healthcare (**c**), indicating mean healthcare use with 95% confidence intervals (Y axis) for each unit increase in the WI-6 score (X axis)

Healthcare use increased with increasing HA for both primary healthcare (Fig. 1a) and somatic healthcare (Fig. 1b). However, there was more uncertainty regarding the association between HA and mental specialist healthcare use (Fig. 1c) due to the few users of this health service. Table 4 presents the results of the regression analyses, presented as the ORs associated with each one-unit change in the WI-6 score.

**Table 4:** Unconstrained-continuation ratio regression models for healthcare utilisation (primary, somatic specialist and mental specialist healthcare). Odds ratio (OR) and 95% confidence interval (CI) shown for the exposure variable health anxiety

Outcome variable		OR (95% CI) represents:	Unadjusted model		Adjusted for physical and mental^**a**^ morbidity		Fully adjusted model^b^	
			OR	95% CI	OR	95% CI	OR	95% CI
Primary healthcare, N: Unadjusted: *n* = 18,499, Adj. morbidity: *n* = 18,249 Fully adjusted: *n* = 16,603	Non-use		–		–		–	
	1st level of use	*1st* vs *non-use*	1.07**	1.05–1.08	1.05**	1.03–1.07	1.07**	1.05–1.09
	2nd level of use	*2nd* vs *1st level or lower*	1.10**	1.08–1.11	1.07**	1.06–1.09	1.08**	1.06–1.10
	3rd level of use	*3rd* vs *2nd level or lower*	1.14**	1.12–1.15	1.10**	1.09–1.12	1.11**	1.09–1.13
	4th level of use	*4th* vs *3rd level or lower*	1.20**	1.18–1.21	1.15**	1.14–1.16	1.15**	1.14–1.17
Somatic specialist healthcare, N: Unadjusted: *n* = 18,311, Adj. morbidity: *n* = 18,073 Fully adjusted: *n* = 16,389	Non-use		–		–		–	
	1st level of use	*1st* vs *non-use*	1.06**	1.04–1.07	1.04**	1.03–1.06	1.05**	1.03–1.06
	2nd level of use	*2nd* vs *1st level or lower*	1.09**	1.08–1.11	1.07**	1.06–1.09	1.09**	1.07–1.10
	3rd level of use	*3rd* vs *2nd level or lower*	1.10**	1.08–1.12	1.07**	1.05–1.09	1.08**	1.06–1.11
	4th level of use	*4th* vs *3rd level or lower*	1.16**	1.14–1.17	1.13**	1.11–1.14	1.14**	1.12–1.16
Mental specialist healthcare, N: Unadjusted: *n* = 18,158, Adj. morbidity: *n* = 16,636 Fully adjusted: *n* = 15,142	Non-use		–		–		–	
	1st level of use	*1st* vs *non-use*	1.15**	1.12–1.19	1.10**	1.06–1.14	1.07**	1.03–1.12
	2nd level of use	*2nd* vs *1st level or lower*	1.13**	1.08–1.17	1.06*	1.02–1.12	1.06*	1.01–1.11
	3rd level of use	*3rd* vs *2nd level or lower*	1.15**	1.11–1.19	1.07*	1.02–1.12	1.05*	1.01–1.11
	4th level of use	*4th* vs *3rd level or lower*	1.20**	1.16–1.24	1.09**	1.05–1.14	1.07*	1.02–1.13

* Significant below 0.05 level

** Significant below 0.01 level

^a^ mental illness is registered with the question “have you had mental illness for which you have sought help?” when analysing primary and somatic specialist healthcare use, and with HADS for mental healthcare use

^b^ Included adjustment variables: Age, gender, education, household income, physical and mental morbidity, living with a spouse/partner, quality of friendship and participation in organised activity

An increased WI-6 score was positively associated with increased utilization of all three health services (Table 4). When we adjusted for confounders, including physical and mental morbidity, the association remained significant, and the OR was only marginally reduced.

### The impact of health anxiety was larger with higher healthcare use

In primary and somatic specialist healthcare, the odds of increased use increased progressively with each one-point increase in the WI-6 score, indicating that the impact of HA was more prominent with higher healthcare use.

In the fully adjusted model, a one-point increase in the WI-6 score resulted in a 7% increased odds of the lowest level of use of primary healthcare compared to non-use. Furthermore, with a one-point increase in the WI-6 score, the OR of the highest level of use compared to all lower levels increased to 1.15.

In somatic specialist healthcare, a one-point increase in the WI-6 score resulted in a 5% increased odds of the lowest level of use compared to non-use. Similar to primary healthcare use, we found a progressive pattern in the OR of somatic specialist healthcare use, with a one-point increase in the WI-6 score increasing the OR of the highest level of use compared to all lower levels to 1.14.

Only 4% of the participants reported using mental specialist healthcare in the past 12 months. A one-point increase in the WI-6 score resulted in a 7% increased odds of use compared to non-use and a significant increase in the OR of a higher level of use compared to all lower levels. The association between HA and mental specialist healthcare use did not show the progressive pattern seen in primary and somatic specialist healthcare.

### Adjusting for confounders, especially physical and mental morbidity, hardly affected the association between health anxiety and healthcare use

The association between HA and primary healthcare use and somatic specialist healthcare use remained nearly unchanged for the first level of use compared to that for non-use after adjustment for the confounders; after adjustment, the OR remained at 1.07 in primary care and changed from 1.06 to 1.05 in somatic specialist healthcare.

For higher levels of use, adjustment for the confounding variables only slightly reduced the effect measure for the OR for both primary and somatic specialist care use and was largest for primary healthcare use; the OR changed from 1.20 to 1.15 for the fourth level of use compared to all lower levels for primary care, whereas the OR changed from 1.16 to 1.14 for somatic specialist healthcare. The association between mental specialist healthcare use and HA was more affected by adjustment for the confounding variables; however, these results should be interpreted with caution due to few participants in the user groups compared to non-users.

### Interaction between the WI-6 score and morbidity

We found a significant interaction between HA and physical illness regarding primary healthcare use, and stratified analyses are presented in Supplementary Table 1. For the participants reporting multimorbidity, the WI-6 score was not associated with the lower levels of use of primary healthcare (1–2 consultations), but a one-point increase in the WI-6 score resulted in a significant increase in the odds of a higher level of use compared to all lower levels.

There was also a significant interaction between the WI-6 and HADS scores that affected the use of mental specialist healthcare (Supplementary Table 2). Due to the few participants with HADS scores ≥15 points (*N* = 193) distributed in the different levels of mental specialist healthcare use, only an unadjusted analysis was performed. In the unadjusted model, the WI-6 score was significantly associated with the first level of use compared to non-use for those with HADS scores ≥15 but was not significantly associated with increased levels of use.

## Discussion

### Main findings

In our study, we found that HA was independently and positively associated with the utilization of primary, somatic specialist and mental specialist healthcare. This significant association remained after we adjusted for confounders, including physical and mental morbidity. For all three health services, a one-point increase in the WI-6 score significantly increased the odds of a higher level of use compared to all lower levels. Although the magnitudes are relatively small, the estimates show how even a very small increase in health anxiety is associated with increased level all healthcare use. To our knowledge, we are the first to report that HA, as a continuous construct, in a general population is significantly associated with increasing levels of use of different healthcare services. This finding implies that lower levels of HA should also be recognized. Interestingly, the trends in these associations were similar across all health services, in contrast to our hypothesis.

### Primary and somatic specialist healthcare use

Primary healthcare use largely occurs through self-initiated contact, and we found that increased levels of HA were associated with increasing levels of consultations. In accordance with other studies [20, 23, 28], this association was not altered by adjustment for mental or physical illness. However, we found a significant interaction between HA and the number of illnesses (Supplementary Table 1). For the participants with two or more present chronic illnesses, the association between HA and primary care use was not significant for infrequent users (1 or 2 consultations per year). Many patients with multimorbidity often have one or two consultations a year as an arrangement with their GPs and not as self-initiated contact, and their use may therefore not be triggered by HA.

The demand for service in specialist healthcare in Norway is predominantly driven by referrals from primary care, and it was therefore surprising that we found such a strong association between HA and all levels of somatic specialist healthcare use. The association between HA and somatic specialist healthcare use has been previously documented in patients [21, 22], but there have been few population studies. Bobevski et al. [28] reported that people with severe HA were more likely to use specialist medical services (OR 1.7) and to be frequent attenders in somatic specialist healthcare (OR 2.4). However, our results shown in Table 4 demonstrate that lower levels of HA were also positively associated with somatic specialist healthcare use. This finding is supported by Hansen et al. [39], who found a dose-response association of HA and healthcare use in patients recruited from a hospital setting. Studies conducted in somatic specialist healthcare have reported a prevalence of severe HA among patients as high as 20–60% [22, 40], indicating that among those who use health services, HA is common.

### Mental specialist healthcare

The association between HA and mental specialist healthcare use has been less examined. However, an Australian survey with 8841 participants aged 16–85 included questions concerning mental healthcare use. Based on this survey, Sunderland et al. [23] reported healthcare use due to a mental health problem, and Bobevski et al. [28] assessed whether HA was associated with healthcare use and explored high-frequency use of mental health services. Both studies reported that people with current HA, as a dichotomous construct, used mental healthcare more than those without HA. Bobevski et al. [28] found that HA was associated with increased odds of mental healthcare use but not with a higher frequency of use. We found significantly increased odds of higher use with even a small increase in HA. Our results should be interpreted as preliminary findings due to the relatively small proportion of participants who had used mental health services; however, we are the first to highlight that HA may also be an important factor for the frequency of consultations in mental specialist healthcare.

### Strengths and limitations

One major strength of this study is the large representative sample, which enabled us to include users of different health services and non-users. The large study sample made it possible to explore different levels of healthcare use and examine HA as a continuous construct and therefore to assess healthcare use with increasing HA. The magnitude of healthcare use reported in our study is close to reports of national healthcare use [41, 42], indicating that the study participants are representative of the Norwegian population.

In the survey, the introduction to the HA questionnaire (“In the past 12 months, have you…”) was omitted. This limited our knowledge of the time frame that the participants used as a reference. For people with established severe HA, HA shows little or no variation over time [13], but there is insufficient knowledge about the time variation in lower levels of HA.

All our results are based on self-reports, which are prone to recall bias. A Norwegian survey [43] found overall close agreement between self-reports regarding morbidity and medical records, with a tendency for under-reporting in self-report measures. Additionally, healthcare utilization has been found to be under-reported in self-reports, especially with increased healthcare use and in older age [44]. If under-reporting was a factor in this study, the strength of the observed association may have been under-estimated.

Because of our cross-sectional design, we cannot conclude whether HA is the cause of increased healthcare use or a consequence of use. However, prospective studies have found that HA is an independent predictor of future healthcare utilization, independent of morbidity [31, 39]. As there is an increase in healthcare use even with low levels of HA (Fig. 1) and independent of morbidity, the association can hardly be explained by experiences of the healthcare system. We therefore believe that HA was a driver of healthcare use in our study.

### Impact of HA on healthcare utilization

Most people with severe HA contact their GPs rather than other health personnel [18]; however, HA is often unrecognized since the patient’s somatic complaints dominate the clinical encounter. Severe HA is a persistent condition if left untreated, and misguided treatment, screening and reassurance from somatic healthcare might not reduce or might even trigger underlying anxiety rather than treat it [18]. The mean level of HA in our population was 3.1 with a mode of 0, indicating that the majority of participants had low levels of HA. This may be interpreted as a normal attitude towards own health. However, even the lower HA scores were associated with increased healthcare use. All questions included in the WI-6 indicates a negative value, and there is no evidence that a lower score indicate health negligence. When background illness is accounted for, all use initiated by health anxiety can be considered overuse. For healthcare services overall, this association contributes to a large number of consultations per year.

Only a small proportion of the participants in our study were frequent users of specialist health services. However, 25% of the participants had 1–2 consultations with somatic specialist healthcare (Table 2). HA seems to be an important driver of these consultations. Although GPs maintain that medical reasons are the main reason for referrals, a significant number of referrals are provided to reassure the patient [2]. This finding is in accordance with our results, indicating that HA is an independent driver of healthcare use in specialist health services. The increased use of specialized healthcare with increasing HA may raise the risk for over-diagnosis and over-treatment and inappropriate use of healthcare, especially in somatic specialist care. Optimally, lower levels of HA in patients should be recognized and dealt with in primary healthcare. If HA assessment fails and patients are referred, the consequences for both patient and specialist care may be large. This study indicates that also lower levels of HA should therefore be of increased focus in patient consultations.

This study makes an important contribution to the research field of HA, in which most studies have explored HA as a dichotomous condition that is either severe or non-existent. Our results support previous research showing a dose-response association between HA and healthcare use [39] and suggest that not only severe HA is severe.

## Conclusions

Our study demonstrates that HA, as a continuous construct, was significantly and positively associated with the utilization of primary, somatic specialist and mental specialist healthcare in an adult general population. One small increase in HA was associated with progressively increased healthcare use across the three health services, indicating that the impact of HA is more prominent for higher healthcare use.

## Supplementary Information

**Additional file 1.**

**Additional file 2.**

## Data Availability

All data are available by applying to the Tromsø Study: https://uit.no/research/tromsoundersokelsen

## Abbreviations

HA: Health anxiety
WI-6: Whiteley Index-6
GP: General practitioner
OR: Odds ratio
PHC: Primary healthcare
SSHC: Somatic specialist healthcare
MSHC: Mental specialist healthcare
HADS: Hospital Anxiety and Depression Scale
GBP: British pound sterling
NOK: Norwegian kroner
