# Supplementary material for: Health anxiety is an important driver of healthcare use
Source: BMC Health Serv Res. 2022 Feb 2;22:138. doi: 10.1186/s12913-022-07529-x (PMC8812228; doi:10.1186/s12913-022-07529-x)
Supplement: Supplementary file 1 — Additional file 1. [file 12913_2022_7529_MOESM1_ESM.docx]

Supplementary Table 1: Associations between health anxiety and primary healthcare use, stratified by the presence of present physical illness

|  |  | **If physical illness: None** | | | | **If physical illness: One** | | | | **If physical illness: Two or more** | | | |
| --- | --- | --- | --- | --- | --- | --- | --- | --- | --- | --- | --- | --- | --- |
|  |  | **Unadjusted** | | **Fully adjusted model^b^** | | **Unadjusted** | | **Fully adjusted model^b^** | | **Unadjusted** | | **Fully adj. model ^b^** | |
| **Outcome variable** |  | OR | †95 % CI |  |  | OR | †95 % CI |  |  |  |  | OR | †95 % CI |
| Primary healthcare | Non-use | - |  | - |  | - |  | - |  | - |  | - |  |
|  | 1^st^ level of use | 1.07** | 1.05 - 1.09 | 1.07** | 1.04 - 1.10 | 1.04* | 1.00 – 1.08 | 1.05** | 1.02 - 1.10 | 1.02 | 0.96 - 1.08 | 1.04 | 0.97 - 1.10 |
|  | 2^nd^ level of use | 1.10** | 1.08 - 1.12 | 1.11** | 1.08 - 1.13 | 1.07** | 1.05 – 1.10 | 1.08** | 1.04 - 1.11 | 1.02 | 0.98 - 1.05 | 1.03 | 0.98 - 1.07 |
|  | 3^rd^ level of use | 1.15** | 1.13 - 1.16 | 1.14** | 1.12 - 1.17 | 1.09** | 1.07 – 1.12 | 1.10** | 1.07 - 1.13 | 1.09** | 1.06 - 1.12 | 1.09** | 1.05 - 1.12 |
|  | 4^th^ level of use | 1.22** | 1.20 – 1.24 | 1.20** | 1.17 - 1.23 | 1.16** | 1.14 – 1.18 | 1.15** | 1.13 - 1.18 | 1.11** | 1.08 - 1.14 | 1.10** | 1.07 - 1.13 |

* Significant below 0.05 level
** Significant below 0.01 level
^b^ Included adjustment variables: Age, gender, education, household income, physical and mental morbidity, living with a spouse/partner, quality of friendship and participation in organised activity
