# Supplementary material for: Health anxiety is an important driver of healthcare use
Source: BMC Health Serv Res. 2022 Feb 2;22:138. doi: 10.1186/s12913-022-07529-x (PMC8812228; doi:10.1186/s12913-022-07529-x)
Supplement: Supplementary file 2 — Additional file 2. [file 12913_2022_7529_MOESM2_ESM.docx]

Supplementary Table 2: Unadjusted association between health anxiety and mental specialist healthcare use, stratified by HADS score

|  |  | **If HADS score below 15 points** | | **If HADS score ≥15 points** | |
| --- | --- | --- | --- | --- | --- |
|  |  | **Unadjusted model** | | **Unadjusted model** | |
| **Outcome variable** |  | OR | †95 % CI | OR | †95 % CI |
| Mental specialist healthcare | Non-use | - |  | - |  |
|  | 1^st^ level of use | 1.12** | 1.07 - 1.17 | 1.08* | 1.02 – 1.15 |
|  | 2^nd^ level of use | 1.10** | 1.04 - 1.17 | 1.01 | 0.94 – 1.08 |
|  | 3^rd^ level of use | 1.11** | 1.05 - 1.18 | 1.01 | 0.95 – 1.08 |
|  | 4^th^ level of use | 1.15** | 1.08 – 1.22 | 1.04 | 0.98 - 1.10 |

* Significant below 0.05 level
** Significant below 0.01 level
